# Chitosan-Based Materials as Effective Materials to Remove Pollutants

**DOI:** 10.3390/polym17182447

**Published:** 2025-09-10

**Authors:** Anathi Dambuza, Pennie P. Mokolokolo, Mamookho E. Makhatha, Motshabi A. Sibeko

**Affiliations:** 1 Department of Chemistry, University of the Free State (QwaQwa Campus), Kestell Road, QwaQwa, Phuthaditjhaba 9866, South Africa; 2028273197@ufs4life.ac.za (A.D.); mokolokolopp@ufs.ac.za (P.P.M.); 2 Department of Metallurgy, University of Johannesburg, Doornfontein Campus, 55 Beit St, Doornfontein, Johannesburg 2028, South Africa; emakhatha@uj.ac.za

**Keywords:** chitosan, pollutants, dyes, heavy metals, adsorption

## Abstract

Chitosan is a natural polymer derived from chitin through the deacetylation process. It has emerged as a key ingredient in sustainable wastewater treatment, due to its biodegradability, non-toxicity, and low cost. This biopolymer possesses abundant functional groups, such as -NH_2_ and -OH, that efficiently interact with pollutants. This review offers a comprehensive evaluation of pollutant separation techniques involving chitosan-based materials, including adsorption, membrane filtration, flocculation, and photocatalysis. It further examines the underlying adsorption mechanisms, emphasizing how pollutants interact with chitosan and its derivatives at the molecular level. Special focus is given to various modifications of chitosan, alongside a comparative assessment of different chitosan-based adsorbents (hydrogels, nanoparticles, nanocomposites, microspheres, nanofibers, etc.), highlighting their performance in removing heavy metals, dyes, and emerging organic pollutants. The reviewed performance of these polymeric materials from 2015–2025 not only gives an insight about the recent advancement but also points the need for the design of high-performing chitosan-based adsorbents with applications in real water matrices.

## 1. Introduction

Global sustainability means managing the available resources so that they meet the needs of present and future generations. A key component of this goal is ensuring access to safe and potable water. Water scarcity remains a global challenge that threatens the well-being of individuals, communities, and the ecosystem. This shortage of water affects not only households but also businesses, agriculture, and various industries [1,2]. In 2015, the United Nations forwarded specific policy targets, the Sustainable Development Goals (SDGs). Among the 17 SDGs objectives, SDG-06 has a main focus on ensuring universal access to clean water and proper sanitation for everyone in 2030 [3].

**Figure 1:**
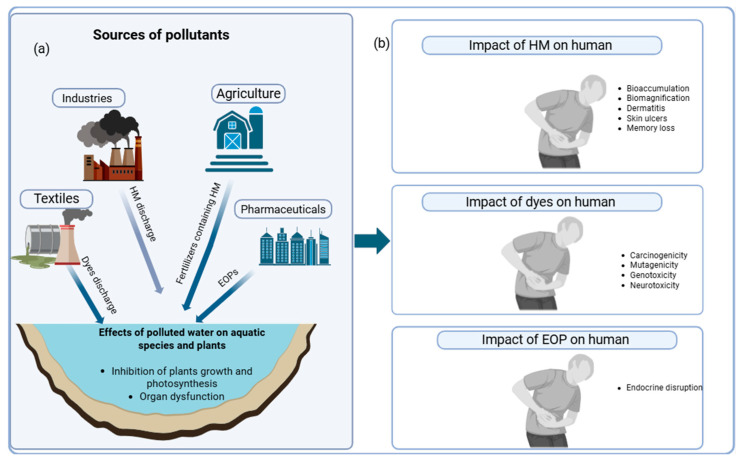
Effects of pollutants, (**a**) Sources of different pollutants and their effects on plants and aquatic species (**b**) Health implications of different pollutants on humans.

Water pollution can arise from natural and human-induced sources. Natural causes include geological processes, climate change, and water–rock interactions [4]. The most common water contamination is through anthropogenic activities (caused by human activities), which include industrial operations, agricultural practices, inappropriate waste disposal, oil spills, and an insufficient sewage system [5]. The most concerning categories of these pollutants are heavy metals, dyes, and emerging organic pollutants (EOPs) [6]. See Figure 1a.

Heavy metals are a group of metals or metalloids that are generally toxic. Certain heavy metals, such as zinc, iron, nickel, etc., are essential for plant and animal development, but when concentrations exceed permissible limits, they become hazardous [7]. A few of the most frequent heavy metals that contaminate the environment include mercury, cadmium, arsenic, chromium, nickel, copper, and lead. As shown in Figure 1b, these Heavy metals are responsible for memory loss, tremors, cognitive dysfunction, kidney dysfunction, skin lesions, skin ulcers, dermatitis, and respiratory issues [8,9,10,11].

The primary source of these hazardous metals in surface water is wastewater effluent from traditional wastewater treatment, the sludge from municipal domestic wastewater that contains household products, and solely household waste [12,13,14]. Toxic heavy metals are introduced into river water directly or indirectly, mostly from industrial waste, municipal and urban influences, and the penetration of polluted surface water into the groundwater aquifer system due to soil and land-use patterns [15]. These heavy metals are resistant to decomposition and can pose serious health implications through bioaccumulation and biomagnification [16].

One of the most polluting industries is the textile industry, which produces a major class of pollutants called synthetic dyes. They are, but not limited to, methylene blue, Congo red, and malachite green, etc., and are complex aromatic structures, often recalcitrant and toxic [17,18]. It is estimated that about 7 × 10^7^ tons of synthetic dyes are annually produced globally, with over 10,000 tons of dyes used by textile industries [19], and about 10% of these dyes are lost during processing, which are then released to the environment [20]. Dyes, even at low concentrations, impact the color of the water bodies, inhibit plant growth and photosynthesis, and are carcinogenic [21,22,23,24]. In addition, there are EOPs, which have detrimental impacts on the environment and human health and lack established emission and environmental monitoring standards [25]. EOPs include pharmaceutical and personal care products, endocrine-disrupting compounds, and persistent organic pollutants [26,27]. Some of the effects of dye and EOP exposure in humans are mentioned in Figure 1b.

The implementation of conventional wastewater treatment technology has been in practice for many years. The treatment process aims to eliminate contaminants from water by removing solids, organic matter, nutrients, and chemical pollutants through three separate treatment stages. Primary treatment involves the physical separation of large particles from water, secondary treatment involves the use of aerobic and anaerobic treatment, then the tertiary stage uses coagulation, membrane filtration, reverse osmosis, flocculation methods, etc. [28]. These methods are limited by factors such as high cost, use of specialized equipment, incomplete removal of dyes, and generation of toxic residues, leading to secondary pollution. As a result, there is an interest in more sustainable, efficient, and environmentally friendly solutions. Adsorption has significantly gained attention as a leading alternative, due to its ability to eliminate a wide variety of pollutants, cost effectiveness, and simplicity [29,30].

**Figure 2:**
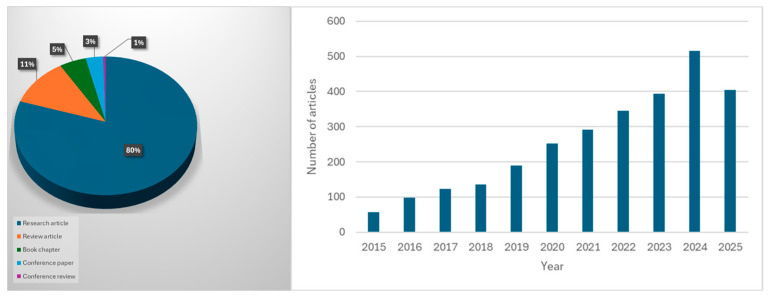
Number of published articles on “chitosan in wastewater treatment” based on the Scopus database (accessed on 14 July 2025).

Adsorption is the process used in wastewater treatment where the pollutants adhere to the surface of the material. Traditional adsorbents (e.g., activated carbon) often face challenges such as high cost, limited reusability, and low selectivity [31]. Natural adsorbents have been utilized in wastewater treatment from cellulosic materials such as grass waste, pomegranate peel, and cellulosic oil palm shell for the effective removal of methylene blue and cupric ions. However, among these diverse arrays of biosorbents, chitosan-based materials have emerged as one of the most promising biopolymers in wastewater treatment [32,33,34,35,36]. Chitosan’s distinctive advantages in water purification stem from its abundant amino and hydroxyl functional groups, which serve to bind strongly with diverse pollutants, including heavy metals and dyes. Additionally, its ability to be chemically and physically modified allows tailoring of properties such as solubility, mechanical stability, and adsorption specificity to suit different treatment needs [37]. According to the retrieval data from the Scopus database (as shown in Figure 2), using the keywords “chitosan AND in AND wastewater AND treatment”, filtering from 2015–2025, 2802 papers, including review articles, research articles, conference papers, etc, have been published, showing the growing interest in studying and utilizing chitosan-based materials in wastewater treatment.

Numerous review articles have focused on the general uses of chitosan or chitosan-based materials in wastewater treatment [38,39,40,41,42,43], its preparation and application for solar step generators [44], its application for the removal of pharmaceutical contaminants [45], its application in the removal of antibiotics [46], its modification and application in retardants [47], and also as an alternative to reduce plastic dependency [48]. This review presents a holistic and critical comparison of multiple sustainable and conventional pollutant removal techniques, pollutant–chitosan-based material interaction mechanisms, and advanced modification techniques of chitosan-based materials, highlighting their potential as versatile and eco-friendly alternatives for efficient contaminant removal.

## 2. Chitosan: Structure, Sources, and Properties

Chitosan is a versatile biopolymer derived from chitin and it is a linear polymer of β-(1→4)2-amino-2-deoxy-d-glucose units (d-glucosamine). The primary sources of chitosan include crustaceans, insects, and fungi. In crustaceans, chitosan is traditionally extracted from the exoskeletons of crabs, shrimp, and lobsters. This method, while common, can be expensive due to the complex extraction processes involved [49]. Insects, including different orders like Lepidoptera and Coleoptera, provide a renewable and abundant source of chitin and chitosan. Additionally, the extraction from insects is gaining attention due to its lower environmental impact and high yield potential [50]. Fungi, filamentous species to be specific, are significant sources of chitosan. Fungal chitosan is noted for its controllable physicochemical properties, making it suitable for diverse applications in food preservation and biomedicine [51].

**Figure 3:**
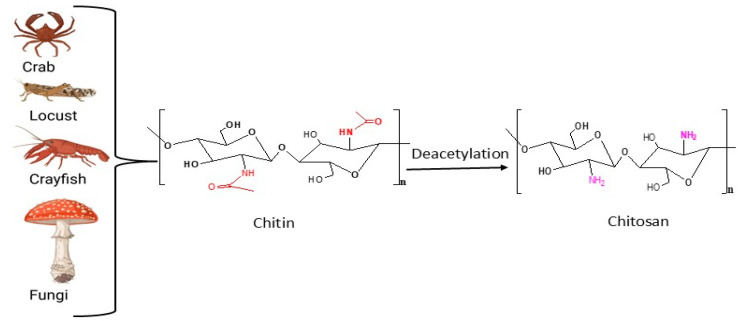
Sources and deacetylation of chitin to chitosan.

The conversion of chitin into chitosan through deacetylation is a significant process. As shown in Figure 3, this process involves the removal of acetyl groups from chitin. Deacetylation can be achieved through various methods, including chemical and enzymatic approaches, each affecting the properties of the resulting chitosan. Chemical deacetylation involves the use of a strong base, such as NaOH. Another key parameter that is worth noting is the consideration of the degree of acetylation and chitosan yield, as studies indicate that varying the mass ratio of chitin to NaOH and the reaction time influences the degree of deacetylation and the chitosan yield [52,53]. Enzymatic deacetylation uses chitin deacetylases (CDAs), for the conversion of chitin to chitosan, however, it is less effective than the chemical method, because CDAs remove a limited number of acetyl groups, leading to a low degree of deacetylation, which in turn does not suffice for chitosan production [54]. It is important to note that fully acetylated or deacetylated chitosan chitin does not exist, but a degree of acetylation of 50% and more is considered a chitosan [55].

The biodegradability and non-toxicity of chitosan make it a preferred candidate in various fields, including wound dressing and wastewater treatment. The degradation of chitosan occurs through two pathways, enzymatic and chemical degradation. The enzymatic degradability of chitosan is facilitated by enzymes such as lysozymes and chitosanase, which break down the polymer into smaller oligosaccharides and later into glucose and glucosamine. Chemical degradation occurs in acidic or alkaline environments, leading to the hydrolysis of chitosan into smaller fragments [56,57]. Despite the properties chitosan possesses, it has some drawbacks that limit its application in wastewater treatment, such as poor solubility in neutral or alkaline pH, low mechanical strength, and poor thermal stability; therefore, modification becomes crucial to overcome those limitations [58,59].

## 3. Modifications of Chitosan

Chitosan is rich in amine and hydroxyl groups, and these groups make it easier for this biopolymer to undergo different modifications, allowing the introduction of new functional groups to the structure, increasing the adsorption capacity of chitosan towards pollutants [60].

### 3.1. Alkylation

Alkylation is one of the most used modifications of chitosan, and this reaction leads to various groups being introduced into the amino sites. For instance, the most widely employed modifications in alkylation are quaternization and Schiff base synthesis. Quaternization modifications primarily convert the primary amino groups into quaternary ammonium salts. Quaternization includes the reaction of the amino group of chitosan and halogenated hydrocarbon, enhancing the positive charge of chitosan, and making it soluble in basic conditions. This positive charge enables chitosan to achieve a strong affinity with anions and enhance chelation [61,62]. For instance, Stepnova et al. [61] synthesized alkylated chitosan with quaternary ammonium groups, which had a removal percentage of more than 90% towards arsenate ions. Quaternized chitosan has been used in wastewater treatment to remove chromium ions, cationic dyes, and anionic dyes [63,64,65].

The first chitosan Schiff base (CSB) was synthesized in 1977 through the reaction of chitosan and different aldehydes. Since then, the CSB synthesis has been used in various applications [66]. This Schiff reaction is a unique type of alkylation reaction. Generally, the amino groups in the chitosan polymer react with aldehyde or ketone to form an imine linkage (−N═CH–R). CSBs have been used in wastewater treatment for the removal of Cd (II) and Fe (III) ions [67], Cu (II) ions [68], Pd (II) ions [69], Cr (II) ions [70], Bismarck Brown and Rhodamine B dye [71], methyl green [72], and methyl orange [73].

### 3.2. Acylation

Acylation in chitosan modification is the process of introducing the acyl group to the polymer through the reaction with acyl chloride or anhydrides, forming the amide derivatives. This is performed on the amino group or hydroxyl group, being N-acylation on the amine group and O-acylation on the hydroxyl group. O-acylation is similar to esterification. It is worth noting that the acylation reaction of many types of acid anhydrides preferentially occurs on amino sites as opposed to hydroxyl sites, which also increases the reaction selectivity of acylation modification. The diversity of reactants also significantly enhances the concepts of modification [74,75]. Fujita et al. [76] synthesized EDTA-linked chitosan by N-acylation of chitosan with ethylenediaminetetraacetic acid (EDTA) monoanhydride in acidic to slightly basic conditions, which acted as a flocculent to remove almost 100% of Cu (II), using chelation. Besides acylation, some substitution reactions have been shown to possess the pollutant removal property, see Table 1.

**Table 1:** Other substitution reactions.

Modification	Type of Substitution Reaction	Materials	Introduced Group	Pollutant Removed	References
Thiolation	Nucleophilic substitution	Thiolated chitosan	Introduction of -SH (thiol) groups	Cu (II) and Cd (II)	[77]
Phosphorylation	Nucleophilic substitution or esterification	Phosphorylated chitosan	Introduction of phosphate groups	U (VI) ions	[78]
Sulfonation	Electrophilic aromatic/hydroxyl substitution	Chitosan lignosulfonate	Introduction of -SO_3_H or -SO_3_^−^	Congo red, Cr (VI), and Rhodamine B	[79]

### 3.3. Crosslinking of Chitosan

**Figure 4:**
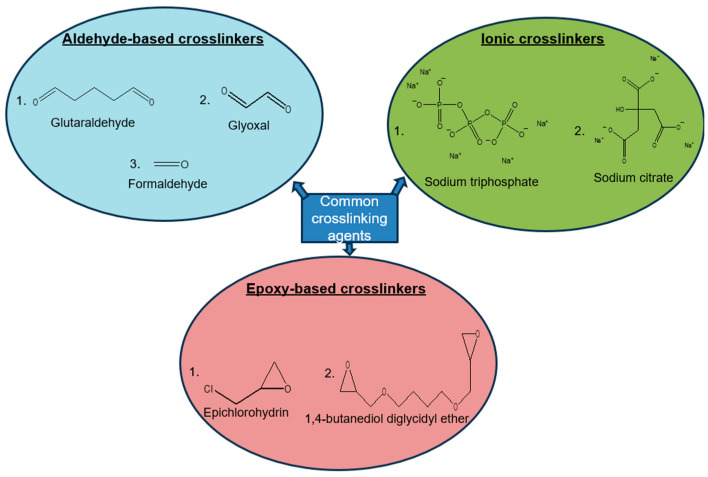
Crosslinking agents of chitosan.

Crosslinking is a stabilizing technique, whereby ionic or covalent bonds between polymer chains are formed, forming a bulky 3D polymer structure, with improved thermal and mechanical properties [80]. This means a crosslinking reaction between chitosan and the crosslinking agent leads to crosslinked chitosan. As shown in Figure 4, there are a variety of crosslinking agents that are compatible with chitosan, including, but not limited to, glutaraldehyde, glyoxal, and formaldehyde [81,82,83]. These are aldehyde crosslinking agents. For instance, glutaraldehyde has demonstrated the ability to crosslink the active amino group of chitosan and bind the molecules of chitosan together with covalent bonds. The electrospun and crosslinked chitosan/poly (vinyl alcohol) (PVA) blend nanofiber has an adsorption capacity of 166.34 mg/g towards Pb (II) ions [84]. There are also epoxy-based crosslinking agents which form covalent crosslinks, such as epichlorohydrin and 1,4-butanediol diglycidyl ether (BDDE) [85,86]. To cite an example, epichlorohydrin crosslinked chitosan was found to be effective in the removal of methyl green dye [87].

There are also ionic crosslinking agents of chitosan, which are specifically designed for electrostatic interactions [88]. Babakhani et al. [89] synthesized a novel ion-imprinted crosslinked chitosan with sodium tripolyphosphate, and the polymeric material has a maximum adsorption capacity of 1.05 mmol/g towards Cd (II) ions in aqueous solution [85,86,87].

### 3.4. Graft Polymerization

Graft copolymerization is an efficient modification process of chitosan. This process involves the reactant’s connection with the functional groups of chitosan, resulting in a polymeric material with dual or merged properties [90]. Graft polymerization can be performed using various monomers such as acrylamide, vinyl acetate, methyl methacrylate, etc. [91]. The grafted chitosan has been reported in many studies to remove Pb (II) ions [92], Cu (II) and Zn (II) ions [93], As (V) ions [94], and methylene blue [95].

### 3.5. Depolymerization of Chitosan

Depolymerization is a critical process in the modification of chitosan for use in wastewater treatment. This can be chemical, physical, or enzymatic, and it breaks down the long chain of chitosan into low-molecular-weight chitosan (LMWC). Unlike the functional group modifications mentioned above, this process solely alters the molecular weight and chain length.

(i) Chemical depolymerization

Chemical depolymerization includes acidic depolymerization, where glycosidic bonds are broken when chitosan’s amino groups are protonated by acidic conditions, such as acetic acid or HCl. This procedure makes chitosan more soluble and lowers its molecular weight [96]. Alkaline depolymerization of chitosan can also use NaOH, which hydrolyzes its polymer chains. However, because of the severe reaction conditions, this method is not as widely used [96]. Furthermore, oxidative depolymerization breaks the glycosidic bonds in chitosan using ozone (O_3_), a potent oxidizing agent [97].

(ii) Enzymatic depolymerization

Enzymatic depolymerization employs enzymes such as chitinases and chitosanases, which break down the glycosidic bond in the polymer chain, resulting in low-molecular-weight oligomers or monomers. As shown in Table 2, this method is more friendly than chemical depolymerization [98,99].

(iii) Physical depolymerization

This depolymerization technique uses physical force to break chitosan chains, such as ultrasonic, ultraviolet, gamma-ray, etc. [100,101]. Table 2 is a comparative analysis of these methods.

**Table 2:** Comparative analysis of depolymerization methods.

Method	Advantages	Disadvantages	References
Chemical depolymerization	Cost-effective, simple, and widely used.	Loss of functional groups such as hydroxyl and amino groups, and environmental concerns due to hazardous byproducts.	[102]
Enzymatic depolymerization	Environmentally friendly, mild reaction conditions, and high specificity.	Limited scalability and high costs.	[98,99]
Physical depolymerization	Chemical-free, simple, and energy-efficient.	Limited control over depolymerization and high energy input.	[103]

## 4. Techniques for Removing Pollutants from Wastewater

There are different techniques that have been used to eliminate pollutants from wastewater, such as flocculation or coagulation, membrane separation, ion exchange, membrane filtration, etc. Each technique offers a distinct advantage in removing different types of pollutants from wastewater.

### 4.1. Flocculation

Flocculation is the process of removing suspended solids or contaminants from a system. This technique involves two mechanisms, such as charge neutralization and bridging, whereby the chitosan-based material will act as a cationic polyelectrolyte, due to its -NH_2_ groups, which in turn neutralize the negative charges on suspended particles, causing their aggregation into massive flocs, which can be removed easily from wastewater [104,105]. In the sweep flocculation mechanism, the chitosan-based material forms a network that captures and settles the suspended contaminants [106]. Wei et al. [107] designed a pH-responsive chitosan-based flocculant, which yielded the maximum flocculation capacity of 1.5 g/g towards Reactive Brilliant Red K-2BP (azo dye) and a high dye removal efficiency of approximately 98.5% under optimized conditions. Another extensive study by Sun et al. [108] prepared a novel chitosan-based flocculent, called carboxylated chitosan-graft-poly[acrylamide-2-acrylamido-2-methylpropane sulfonic acid], with chromium and nickel removal rates of 94.6% and 99.4%, respectively.

### 4.2. Photocatalysis

Photocatalysis is the process that is mostly used in wastewater treatment, which breaks down pollutants under light irradiation. Traditional photocatalysts, such as TiO_2_ and CdS suffer from a small surface area, high band gap, poor adsorption capacity, and poor carrier life [109,110]. The incorporation of these metal oxides with chitosan enhances catalytic activity by providing more active sites and suppressing electron–hole recombination rates [109,110]. In a study conducted by Nyamiati et al. [111], chitosan-TiO_2_ membranes demonstrated effective degradation efficiency of 91.89% towards Pb and Cd in batik waste. Additionally, Ali et al. [112], prepared a novel chitosan-encapsulated metal selenide photocatalyst, which degraded Bromothymol Blue dye by 97% within 100 min under optimized conditions. The FeNi_3_/chitosan/BiOI nanocomposite also showed effective degradation of metronidazole of 100% [113].

### 4.3. Membrane Filtration

Membrane filtration is a separation technique whereby a semi-permeable membrane is used to remove pollutants from wastewater. Chitosan-based materials have been used to remove pollutants from wastewater with this technique. For instance, Machodi et al. [114] synthesized polyethersulphone/chitosan membranes coated with polyamide, which exhibited significant improvement of 56 to 93 L/m^2^.hr in pure water flux upon the addition of 1 wt% chitosan. Mn^2+^, Fe^2+^, Mg^2+^, and Ca^2+^ had rejection percentages of 90.4%, 88.3%, 89.3%, and 75.7%, respectively. Furthermore, in a related study by Bassyouni et al. [115], chitosan nanofiltration membranes fabricated via solvent casting achieved 100% rejection of Direct Blue 78 dye with a permeation flux of 9.3 L/m^2^.h and maintained antifouling performance over 10 h of continuous filtration.

## 5. Adsorption Mechanisms of Chitosan-Based Materials

Understanding the adsorption mechanisms of chitosan-based materials is crucial. As mentioned earlier, chitosan has many functional groups that enable it to interact with pollutants through different adsorption mechanisms. Chitosan can be modified to better suit these mechanisms in wastewater treatment. These mechanisms include, but are not limited to, hydrogen bonding, chelation interactions, and electrostatic interactions.

### 5.1. Electrostatic Interactions

**Figure 5:**
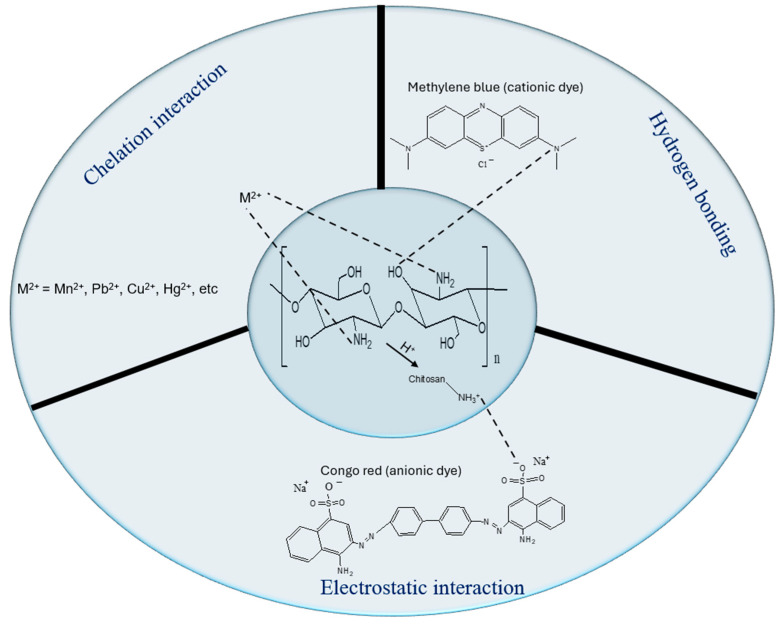
Common adsorption mechanisms of chitosan-based materials for the elimination of dyes or heavy metals.

Electrostatic interaction is a prevalent adsorption process utilized by chitosan-based polymers for the removal of persistent heavy metals, dyes, and EOPs from wastewater. This mechanism arises when charged species are separated by a certain distance due to ionization. The repulsive forces among like charges and the attraction force between unlike charges are noted as a key phenomenon [116]. As shown in Figure 5, the amine group in chitosan is protonated at low pH and becomes more cationic, leading to the attraction of anionic dyes, e.g., azo dyes. This interaction between chitosan and anionic pollutants leads to enhanced binding efficiency and significant pollutant removal rates [117].

### 5.2. Hydrogen Bonding

Hydrogen bonding is a dipole–dipole interaction between molecules. This dipole–dipole contact arises from the covalent bond between hydrogen and two strongly electronegative atoms, such as nitrogen and oxygen in chitosan-based material. Hydrogen bonding interaction between two atoms with a higher affinity for electrons is necessary for the hydrogen bonding interactions. In this case, the link is stronger than the van der Waals force but weaker than an ionic or covalent bond [118]. For instance, the hydrogen bonding mechanism in chitosan-based materials has been described in the elimination of EOPs (phenols and rotenone) [119,120,121] and dyes [122,123,124]. In a study by Kyomuhimbo et al. [125], the general mechanism for the removal of Bismarck Brown (BB), Orange G (OG), Brilliant Blue G (BBG), and Indigo Carmine (IC) dyes was through hydrogen bonding.

### 5.3. Chelation Interaction

Chelation is a process in which a molecule, such as a polydentate ligand with two or more donor atoms, binds to a metal ion to form a chelate complex (see Figure 5). In the case of water remediation, chitosan-based materials have been utilized to remove many ions in wastewater, as they contain many amino groups with donor atoms [58]. Enhanced interactions are essential for an effective adsorption process [126]. Conversely, fluctuations in pH may modify the adsorbent’s structure and disrupt the adsorption process [127]. Lyu et al. [128] synthesized chitosan-Mg-Al layered double hydroxide (CS-LDH) and used it to remove Pb (II) ions, with XPS analysis of CS-LDH conducted before and after the removal procedure illustrating the chelation mechanism. There are numerous studies where this mechanism was involved in the elimination of Pb (II), Cu (II), Co (II), and Cd (II) ions [117,129,130,131].

### 5.4. π–π Interactions

π–π interactions, or stacking, are non-covalent interactions between aromatic rings, and this mechanism is very important in dye removal. However, chitosan lacks an aromatic ring; therefore, to facilitate this mechanism, chitosan must be functionalized with aromatic moieties. Dyes with multiple aromatic rings, such as Methylene Blue and Congo Red, exhibit enhanced π–π interactions due to their extensive π systems. The interaction is further enhanced when the dye molecules are planar, allowing for optimal overlap with the aryl-functionalized chitosan surface [132,133]. The intensity of π–π interactions can be adjusted by using dyes that contain electron-donating groups (e.g., hydroxyl or amino) or electron-withdrawing groups (e.g., sulfonic acid). For instance, protonated chitosan surfaces, where electron-rich regions promote π–π stacking, interact strongly with anionic dyes such as tartrazine [134,135]. This mechanism has been observed in aryl-modified chitosan for the removal of dyes, including Acid Red 88, Reactive Orange 16, and Reactive Blue 221 [136,137,138], as well as in phenol removal [120].

## 6. Chitosan as an Adsorbent

Chitosan-based materials exist in various forms, depending on how the material was processed to serve as an adsorbent. These forms include films, nanofibers, membranes, hydrogels, nanocomposites, sponges, and nanoparticles. Each adsorbent form has its own unique advantages; however, according to the literature, the most promising chitosan-based material is hydrogel, due to its high water swelling capacity [139]. To evaluate the efficiency of chitosan-based materials, models of adsorption kinetics are used to elucidate the interaction mechanisms between adsorbents and pollutants (mostly pseudo-first-order and pseudo-second-order kinetic models) [140,141], while adsorption isotherms assess both adsorption efficiency and economic feasibility. Commonly applied isotherm models include Langmuir, Freundlich, Redlich–Peterson, and Sips, each reflecting different surface and interaction characteristics [142]. The following sections detail the preparation and application of these chitosan-based adsorbents in wastewater treatment.

### 6.1. Sponges

Chitosan sponges are mostly created through freeze-drying techniques, which result in a porous structure that enhances adsorption capacity for various pollutants, such as dyes, heavy metals, and EOPs [143], for instance, Xu et al. [144] synthesized xanthate-modified sponge-like chitosan-based adsorbent (CTS-SX) through a facile method, which exhibited excellent adsorption performance for Sr (II) and Cs (I) with maximum adsorption capacities of 76.21 and 133.15 mg/g, respectively. 

### 6.2. Films and Membranes

Chitosan-based films and membranes are mostly prepared by casting and drying a chitosan solution and sometimes modified with crosslinking agents to improve the mechanical strength and stability in acidic conditions [145,146]. These materials are effective in membrane separation processes, notably for nanofiltration and microfiltration, where they help in the removal of heavy metal ions and emulsified oil droplets [145].

### 6.3. Nanofibers, Nanoparticles, and Nanocomposites

Chitosan nanofibers are produced through electrospinning, which enables them to have a large surface area and high porosity, enhancing adsorption capabilities [143]. Chitosan nanoparticles are pure, or nearly pure, chitosan particles at the nano-range, which can be prepared through ionotropic gelation, microemulsion, emulsification diffusion methods, etc. [147]. Chitosan composites are mostly doped with nanoparticles or other materials (e.g., metal oxides, clay, graphene), to improve the adsorption capacity in wastewater treatment [148].

### 6.4. Hydrogels

Hydrogels are the most remarkable chitosan-based adsorbents in wastewater treatment because of their three-dimensional network, which can swell and retain large amounts of water. They are synthesized through the crosslinking of chitosan with aldehyde-based crosslinkers (e.g., glutaraldehyde) [148]. Table 3 highlights the recent advancements and use of these materials in water remediation.

**Table 3:** Recent studies on the use of chitosan-based adsorbents in wastewater treatment and their adsorption capacities.

Chitosan-Based Adsorbent	Pollutant	Reusability	pH	Adsorption Capacity (mg/g)	Mechanism Involved	Kinetic Studies	Isotherm Model	References
Sponges								
ZIF-8@chitosan (ZIF-8@CS) composite sponge	Congo Red	5	-	987.01	Electrostatic interactions, hydrogen bonding, π–π interactions	PFO	LIM and FIM	[149]
PEI-S-CS sponge	Hg (II)	6	1–7	1227.15	Chelation	PSO	LIM	[150]
Chitosan/silver cluster-loaded cellulose nanofibril/Cu-ZIF-8	Cr (VI)	10	2	171.20	Electrostatic interactions, hydrogen bonding	PSO	LIM	[151]
Ammonium-modified chitosan composite sponge	Congo Red	5	6	1261.64	Electrostatic interactions, hydrogen bonding	PSO	SIM	[152]
Chitosan–alginate sponge	Maranth, Carmine, and Sunset Yellow	6	2	94.34, 111.50, and 80.05, respectively	Electrostatic interactions	PSO	LIM	[153]
Microspheres								
Porous magnetic chitosan microspheres (PPy@PMCS)	Cr (VI)	4	2	330.42	Chelation	PSO	LIM	[154]
Chitosan-based composite microspheres (CP)	Cr (VI)	4	3	299.69	Electrostatic interaction, chelation	PSO	LIM	[155]
Chitosan-based composite microspheres (CP)	Eriochrome Black T dye (EBBR)	-	5	317.21	Electrostatic interactions	PSO	LIM	[155]
Nitrilotriacetic acid-modified magnetic chitosan microspheres	Tetracycline (TC)	5	8	625.52	π–π interactions, hydrogen bonding	PSO	FIM	[156]
Iron-doped chitosan microspheres	As (III)	3	8	≥125	Electrostatic interaction, chelation	PSO	FIM	[157]
Nanoparticles								
Magnetic chitosan nanoparticles	Reactive Red 141 (RR-141), Reactive Yellow 14 (RY-14)	5	RR-141: 5.5 RY-14: 5.6	RR-141.00: 98.8 RY-14: 89.70	Electrostatic interactions	PSO	FIM	[158]
Magnetic Fe_3_O_4_-chitosan nanoparticles	Cs (I)	5	-	161.30	Electrostatic interactions	PSO	LIM	[159]
Magnetic thiazole-functionalized chitosan nanoparticles	Cd (II)	5	4	200.00	Ion exchange, chelation	PFO	SIM and LIM	[160]
Magnetite-functionalized chitosan nanoparticles grafted with TDP	Cr (VI)	5	4	299.00	Electrostatic interactions, chelation, ion exchange	PFO	SIM and LIM	[161]
Magnetic ion-imprinted chitosan nanoparticles (MIIP)	Ni (II)	15	7	18.50	Electrostatic interactions, chelation	PSO	LIM	[162]
Nanofibers/membranes								
Electrospun CS/CQDs/PCL nanofiber membrane	Ni (II)	5	6	341.80	Electrostatic interactions, chelation	PSO	LIM	[163]
CS-g-PNVCL/ZIF-8	Phenol	5	3	395.80	π–π interactions, electrostatic interactions	PSO	RPIM	[164]
Electrospun CS/PVA nanofiber loaded with Ce-MOF	Malachite Green (MG)	8	5	359.20	Electrostatic interactions, chelation, hydrogen bonding	PSO	LIM	[165]
Chitosan-sulfonated polyphenylsulfone nanofibers	Congo Red	4	6.5	531.56	Electrostatic interactions, chelation, hydrogen bonding, hydrophobic interactions	PSO	FIM	[166]
Functionalized cellulose/chitosan porous nanofibrous membranes	Cu (II)	6	5	121.06	Electrostatic interactions, chelation	PSO	LIM	[167]
Magnetic ion-imprinted electrospun nanofiber membrane	Pb (II)	7	7	133.20	Ion-imprinting, chelation, electrostatic interactions	PSO	LIM	[168]
Chitosan/Nylon-6 (CS/N) nanofiber	Cu (II)	8	4	240.00	Electrostatic interactions, chelation	PSO	LIM and FIM	[169]
Chitosan–lignin composite cast membrane	MB	5	7	241.62	Hydrogen bonding, electrostatic interaction, van der Waals forces	PFO	LIM	[170]
Films								
ZnFe_2_O_4_/HEC/chitosan film	Methyl Orange	5	8	914.00	Electrostatic interactions and hydrogen bonding	PSO	LIM	[171]
CS/PVP/β-CD/NCC composite film	Cu (II)	5	5	148.20	Electrostatic interactions, hydrogen bonding, ion exchange, surface complexation	PSO	LIM	[172]
CMC-chitosan film	Pb (II), Cd (II)	4	5	Pb (II): 483.00, Cd (II): 123.00	Complexation, electrostatic interactions	PSO	LIM	[173]
Chitosan film	As(V)	4	3	15.23	Ligand exchange, electrostatic interactions	PSO	LIM and FIM	[174]
Chitosan film	Diclofenac (DCF)	10	5	~10.00	Electrostatic interactions	PSO	FIM and TIM	[175]
Nanocomposites								
Fe_3_O_4_–chitosan@bentonite nanocomposite	Congo Red	8	5	169.00	Electrostatic interactions, hydrogen bonding, π–π stacking	PSO	LIM	[176]
Fe_3_O_4_@CS-CGSB	Pb (II), Cd (II)	5	7	Pb: 394.3, Cd: 390.99	Electrostatic interactions and chelation	PSO	FIM	[177]
Crosslinked alginate–rice husk–GO–chitosan nanocomposite	Pb (II)	5	6.5	295.50	Electrostatic interactions, ion exchange, chelation	PSO	FIM	[178]
GO–chitosan nanocomposite	Cr (VI), Ni (II)	4	Cr (VI): 5 Ni (II): 8	Cr: 1.01 Ni: 1.34	Electrostatic interactions, hydrogen bonding	PSO	Cr (VI): FIM Ni (II): LIM	[179]
CS–GO nanocomposite	Rotenone	3	1	92.59	Hydrogen bonding, π–π interactions	PFO	LIM	[121]
Fe_3_O_4_/chitosan/ZIF-8 nanocomposite	Phenol	-	9.91	6.44	Hydrogen bonding, π–π interactions	PSO	LIM	[120]
Hydrogels								
Glucan/chitosan hydrogel	Cu (II), Co (II)	-	7	Cu (II): 342.00, Co (II): 232.00	Ion exchange, electrostatic interactions	PSO	LIM	[141]
Chitosan-based hydrogels	2,4-Dichlorophenoxyacetic acid	5	Neutral (7)	75.29	Monolayer formation and multisite interactions	PSO	LIM	[180]
Sodium alginate/sodium lignosulfonate/carboxylated chitosan/polyethyleneimine composite hydrogels	Anionic (AD) and cationic dyes (CD)	5	-	AD > 550 CD > 1900	Electrostatic interactions, π–π interactions	PSO	LIM	[135]
Carboxymethyl chitosan/sodium alginate hydrogels	Cd (II), Cr (III)	-	-	Cd (II): 314.60 Cr (III): 289.10	Electrostatic interactions, ion exchange	PSO	LIM	[181]
Chitosan-based hydrogel	Tetracycline	4	8	541.30	Electrostatic interactions, hydrogen bonding	PSO	LIM	[121]
Acrolein-crosslinked chitosan hydrogel	Acid Blue 93 (AB93)	12	-	1839.00	Hydrogen bonding, electrostatic interactions	PSO	LIM	[182]
Amine-thiourea modified magnetic chitosan hydrogel	Ce (III)	5	6	156	Chelation, electrostatic interactions	PSO	LIM	[183]
XMPC hydrogel	Cu (II)	5	5–6	185	Chelation, electrostatic interactions	PSO	LIM	[184]

Pseudo First Order (PFO), Pseudo Second Order (PSO), Langmuir Isotherm Model (LIM), Freundlich Isotherm Model (FIM), Sips Isotherm Model (SIM), Redlich–Peterson Isotherm (RPIM), and Temkin Isotherm Model (TIM).

## 7. Conclusions, Future Work, and Recommendations

Chitosan-based adsorbents have emerged as one of the most promising materials in wastewater remediation, as demonstrated across various studies reviewed in this work from 2015–2025. The versatility of chitosan is brought about by its modifiable structure through various methods, which tailor its selectivity and affinity towards specific contaminants such as dyes, heavy metals, and emerging organic pollutants. Sponges have high capacities and rapid uptake from their high porosity, although they have lower mechanical stability. Films and membranes have a continuous filtration due to their superior strength. Nanofibers provide fast adsorption from a large surface area, and magnetic microspheres/nanoparticles enable easy recovery and reuse. These differences reflect how their form of structure affects their performance and application suitability. Among the reviewed chitosan-based adsorbents, hydrogels stand out as the most effective due to their 3D structure and high swelling capacities, leading to more adsorption sites. Most kinetic studies favor the pseudo-second-order model, indicating chemisorption as the main removal mechanism on the reviewed chitosan-based adsorbents, while Langmuir and Freundlich isotherms best describe monolayer and heterogeneous surface adsorption, respectively.

Despite this significant improvement in the utilization of chitosan-based materials in wastewater treatment, some challenges need to be considered before these materials can be used in large-scale water purification systems. (i) The issue of the decrease in adsorption capacity of these materials in a few cycles. (ii) There are few studies that test chitosan-based adsorbent materials in fixed-bed or membrane–adsorption hybrid systems to move closer to industrial application, so integration into a continuous system rather than closed system should be carried out. (iii) These adsorbent materials are tested in controlled laboratories, not in real wastewater matrices, where there can be competitive ions or organic matter interference. So, to bridge this gap, new chitosan-based adsorbents that preserve structural integrity across multiple cycles should be designed; furthermore, there should be an expansion of performance testing to real wastewater matrices.
